# Public Health Emergency Preparedness After COVID‐19

**DOI:** 10.1111/1468-0009.12615

**Published:** 2023-04-25

**Authors:** JOSHUA M. SHARFSTEIN, NICOLE LURIE

**Affiliations:** ^1^ Johns Hopkins Bloomberg School of Public Health; ^2^ Coalition for Epidemic Preparedness Innovations

**Keywords:** public health, emergency preparedness, COVID‐19

## Abstract

**Policy Points**

- The critical task of preparedness is inseparable from the regular work of advancing population health and health equity.

On february 26, 2020, president donald j. trump held a press conference to discuss a novel coronavirus spreading around the world. “Johns Hopkins, highly respected, … they did a study, comprehensive, the countries best and worst prepared for an epidemic,” the president declared from the White House briefing room. “And the United States, we're rated No. 1.”^1^

He was not misinformed. Released in October 2019, the study, known as the Global Health Security Index, was the product of a two‐and‐a‐half‐year effort involving leading emergency preparedness researchers and practitioners from around the world. The United States scored an 83.5 out of 100 points and was top ranked in five of six categories, including prevention, early detection and reporting, rapid response and mitigation, sufficient and robust health system, and compliance with international norms.^2^

A little more than two years after President Trump's press conference, more than one million Americans would be dead from SARS‐CoV‐2 infection. The impact of the pandemic to the US economy would be estimated at more than $16 trillion.^3^ Far from being the most‐ready nation on the planet, the United States has been exposed as especially ill‐prepared for the greatest public health crisis in a century—and the US approach to public health emergency preparedness has come due for reexamination.

Public health emergency preparedness has been defined as “the capability of the public health and health care systems, communities, and individuals to prevent, protect against, quickly respond to, and recover from health emergencies, particularly those whose scale, timing, or unpredictability threatens to overwhelm routine capabilities.”^4^ In this paper, we trace the history of this effort in the United States, from the terrorist attacks of September 11, 2001, to the present. Then, we propose a new vision for the field of public health emergency preparedness for the future, one that is integrally connected to a future vision for public health.

The story is far from simple. Over the past two decades, major investments in the development of countermeasures have led to important successes, most recently the remarkable development of safe and effective COVID‐19 vaccines. Yet at the same time, a persistent gap between the work of emergency preparedness and the essential activities of national and global public health has contributed to multiple failures. It is not the case that by funding emergency preparedness programs, policymakers directly harmed broader public health efforts. Rather, policymakers have invested in preparedness programs without recognizing that they rest on a set of unstated, untested, and untrue assumptions about the nation's core public health capacity.

Facing Ebola, H1N1, COVID‐19, and other threats, the federal government has provided new investments in medications, tests, and vaccines—but generally without necessary support for the infrastructure to deploy these tools rapidly in the United States and around the globe. In states and localities, public health emergency preparedness staff have written many plans and conducted many drills—but generally without the ability to strengthen the core capacity of their agencies to address urgent health problems. Even when funding has surged in response to emergencies, its temporary nature and specific restrictions have made it difficult to leverage for a modern workforce or core information technology systems. For many years, considerations of equity in preparedness grant programs have been focused on “special populations” and not central to the core expectations or program outcomes.

A reassessment of public health emergency preparedness starts with appreciation of the inadequacy of the siloed frameworks and programs that have characterized much of the past two decades. Coming challenges—including infectious diseases, toxic exposures, and climate change—require a renewed commitment to global partnerships. Domestically, effective preparation for health emergencies rests on the foundation of a robust national public health system, devoted to equity, that delivers foundational public health capabilities to protect all Americans, every day.

## The 9/11 and Anthrax Attacks

The modern history of public health emergency preparedness dates from the events of September 11, 2001. Prior to this time, most preparedness activities fell into discrete subject areas, with infectious disease teams countering outbreaks, environmental health programs responding to toxic exposures, and the National Disaster Medical System responding to events such as hurricanes that overwhelmed the health care delivery system. In 1998, President Bill Clinton reportedly read a novel about bioterrorism and pushed for the development of greater domestic response capabilities, including planning for an influenza pandemic and the creation of a stockpile of countermeasures to civilians.^5^ The next year, the Centers for Disease Control and Prevention (CDC) began to fund Centers for Public Health Preparedness at academic institutions, responsible for a broad range of public health training needs, including countering bioterrorism.

The 9/11 attacks on the World Trade Center and the Pentagon, followed soon after by multiple deaths from weaponized anthrax, moved these efforts from the outer edges of political consideration to center stage. Policymakers reacted quickly to strengthen the nation's defenses. This era saw a significant reorganization of the federal government and large investments in countermeasure development and public health programs focusing on bioterror threats.

It was the Bioterrorism Act of 2002, passed by nearly unanimous votes in Congress, that set the modern field of public health emergency preparedness into motion.^6^ The law called on the secretary of the US Department of Health and Human Services (HHS) to create a preparedness program led by a new assistant secretary for emergency preparedness. Congress subsequently appropriated billions of dollars to fill a national reserve of supplies and countermeasures, to upgrade infrastructure at hospitals nationwide, and to support states and localities in purchasing equipment and services, such as interoperable communication systems and syndromic surveillance programs.

Within months, health departments of all sizes were managing a new set of responsibilities, personnel, and funding streams. Training exercises, sometimes led by law enforcement and military personnel, became routine, with a central focus preparing for a bioterrorism event involving smallpox or a large, aerosolized anthrax attack. The CDC's cooperative agreement with states was updated in 2003 to call for more attention to incident management, laboratory connectivity, hospital readiness, smallpox preparedness, laboratory capacity, and mental health services for victims of terrorism.^7^ This funding led to much greater rapid response and incident management capability in many health departments, a focus on emergency communications, and practice for how different levels of government can coordinate in a response.

In all these efforts, however, considerations of equity were secondary. In a section labeled “Other Activities,” the CDC included a paragraph devoted to “special populations” that asked state and local health departments to “describe activities that will be implemented to meet the specific needs of special populations that include but not limited to people with disabilities, people with serious mental illness, minority groups, the non‐English speaking, children, and the elderly.”^8^ This concept of “special populations,” which applied to more than half of Americans, was based around individual “specific needs,” rather than common social determinants that create special vulnerabilities for communities in emergencies.

With the existential threat of bioterrorism capturing the public's imagination, funding public health emergency preparedness became a budgetary priority, even as other public health funding declined. It was not uncommon for health department preparedness programs to have a closet full of expensive new computers and communications devices, lacking interoperability, while other divisions of health departments faced year after year of declining budgets.

This disparity frustrated state and local public health officials. It also reflected the lack of appreciation of the lessons of the anthrax attacks themselves. For example, an October 2003 report by the General Accounting Office, based on extensive interviews with state and local officials, called attention to the “importance of a strong public health infrastructure to serve as the foundation for responses to bioterrorism or other public health emergencies.”^9^

## SARS and Pandemic Influenza

The 2003 outbreak of severe acute respiratory syndrome (SARS) demonstrated how nature's destructive capacity could rival the risk posed by bioterrorism. Yet in 2004, when Congress passed the BioShield Act, the focus of billions of dollars for the development of countermeasures remained on those diseases and exposures determined by the Department of Homeland Security to be bioterrorism threats—including nuclear radiation, smallpox, anthrax, and botulinum toxin.^10^ It was the next year, after the threat of avian pandemic influenza in China made global headlines in 2005, when President George W. Bush and the nation took notice of the risks of a natural pandemic.

The Bush administration and Congress budgeted hundreds of millions of dollars to develop pandemic influenza vaccines, including to create a reserve supply of eggs for vaccine production.^11^ The CDC updated its grant requirements for public health emergency preparedness programs with new expectations for pandemic influenza plans. In 2006, Congress reauthorized the 2002 Bioterrorism Act with the Pandemic and All Hazards Preparedness Act.^12^ This law created the Biomedical Advanced Research and Development Authority, with the mandate and resources to create a national strategy for countermeasure development covering both man‐made and natural threats.

This expansion in mission, however, did not come alongside a stable source of funding for routine public health activities. During the financial crisis of 2008, states and localities dramatically cut back on public health spending, and tens of thousands of public health workers lost their jobs.^13^ The ability of many public health agencies to service their communities deteriorated. Major weaknesses in data systems, workforce, laboratories, and procurement remained unaddressed, within starkly uneven capabilities across the country.^14^ Collaborative work with the health care system was often limited to drills focused on exercising emergency capacities, often overseen by state emergency managers. Alongside the weakening of public health infrastructure was waning national interest in health disparities, a term that the Bush administration tried to eliminate.^15^

## H1N1 Influenza

In early 2009, a novel influenza virus first identified as an H1N1 strain in Mexico began to spread rapidly with early evidence of high acuity among children and teenagers. Within weeks, the World Health Organization declared a pandemic. The ensuing US response reflected both the strengths and weaknesses of preparation efforts to date.

Reflecting large investments in countermeasures, the United States mobilized the reserve supplies of eggs to manufacture large quantities of a pandemic vaccine, which was approved rapidly by the US Food and Drug Administration. HHS paired the vaccine rollout with the use of Centers for Medicare and Medicaid Services (CMS) data to monitor vaccine coverage by region and by race and ethnicity, insurer data to investigate safety signals, and a dedicated approach to crisis and emergency risk communications.^16^ At the state and local levels, health departments distributed supplies and medications from the strategic national stockpiles, relayed key messages on how to stay safe, and oversaw vaccination efforts.

Accompanying these important successes were a series of challenges, reflecting that emergency preparedness funds were not designed to address ongoing weaknesses in core public health capabilities. State and local health departments struggled to track school and work absenteeism, assess local vaccination rates, and respond to concerns about vaccine hesitancy, including among health care workers. Federal agencies had difficulty obtaining needed data across disparate health departments, providing testing supplies at scale, and tracking bed capacity in coordination with health care organizations and clinicians. Other gaps noted in after‐action reports included “a lack of culturally and linguistically appropriate messaging” linked to distrust of vaccine, with recommendations for partnership with “community‐based faith based and grassroots organizations … to reach hard to reach populations.”^17^

From a global perspective, US political leaders did not prioritize sharing vaccine with other nations. The United States held nearly all the contracts for pandemic influenza vaccines, and only after a substantial portion of the US population had been vaccinated did the federal government offer vaccines to the World Health Organization. It then took additional months for vaccines to reach countries in need, by which point the pandemic was subsiding.^18^

In the end, the H1N1 strain was not as dangerous as feared, and the issues identified during the crisis did not elicit major policy responses.

## The 2010s: An Oil Spill, a Nuclear Plant Calamity, Ebola, and Zika

During the decade of the 2010s, a succession of public health challenges illuminated weaknesses in public health emergency preparedness, both domestically and internationally, but again failed to spark major changes.

In April 2010, the *Deepwater Horizon* oil spill led to an environmental and economic disaster in the Gulf Coast. As was the case after the 1989 *Exxon Valdez* oil spill off the Alaska coast, rates of depression, anxiety, suicidality, and substance use in local populations increased.^19^, ^20^ However, underfunded state and local health departments were unable to recognize and act quickly to address the mental health sequelae. Ultimately, HHS launched a disaster distress phone line alongside initiatives to train lay community members in psychological first aid.^21^

Shortly thereafter, another environmental disaster followed. An earthquake and tsunami disabled the Fukushima Daiichi nuclear power plant, leading to the release of radiation in air, water, and soil.^22^ Although the disaster was an ocean and a continent away, it was a warning that disasters know no boundaries. State and local health departments in the United States struggled to find experts for consultation, develop communication materials, and conduct environmental monitoring for radiation, including in water and food. As a result of public anxiety, the US commercial supply of potassium iodide, a radiation countermeasure, was exhausted.^23^ For its part, the CDC was unable to receive standardized and consistent information about radiation levels from health departments across the country.

In 2014, the West African nations of Sierra Leone, Liberia, and Guinea experienced a severe Ebola outbreak, with tens of thousands of fatalities.^24^ After a community case in Dallas, Texas, in a traveler from Liberia, inadequate infection control led to the spread of Ebola to two health care workers, sparking a national panic. The media breathlessly covered the arrival of patients from West Africa who were being evaluated for Ebola.^25^ Hospitals around the country struggled to manage supplies of personal protective equipment and rapidly changing guidance for isolation of suspected cases. Arguments broke out among state and local health departments and their local medical systems over who was responsible for suspected cases, reflecting gaps in communication and collaboration.^26^

Divisive national politics ahead of a pending midterm election further undermined the response.^27^ Governors from across the political spectrum ordered aggressive tracking of immigrants and quarantine enforcement, including with police officers monitoring potentially exposed individuals from their front lawns.^28^ Meanwhile, many state and local health departments were unable to stand up isolation and contact tracing quickly and effectively. Efforts to enlist the support of leaders of African immigrant communities, while uncommon, were helpful.^29^

The Ebola situation tested the national effort to develop countermeasures. Investigational treatments were mobilized for treatment of the few Westerners evacuated from West Africa, but vaccines in development needed to be tested where the outbreak was—in West Africa. By the time study designs were agreed upon and studies were completed, the outbreak had largely subsided. The recognition that an infectious disease elsewhere could rapidly spread to affect Americans led the National Security Council to open a public health office and draft a playbook for a coming pandemic.^30^ However, global coordination efforts to address future similar scenarios proceeded slowly.

In 2015, an unexpected outbreak of Zika virus, a mosquito‐borne disease, began in Brazil and quickly spread through South and Central America to the southern United States.^31^ The virus was found to cause profound damage to fetal development, creating particular challenges in areas without access to abortion services.^32^ Vaccine development started quickly, but federal funding was withdrawn once the epidemic subsided.^33^ Domestically, the US struggled with the basics of the public health response, including reliable surveillance, developing accurate or ample lab testing, implementing science‐based travel advisories and other effective communications, and coordinating mosquito control at the state and local levels.^34^

Zika's broad spread was a warning sign of the convergence of the climate crisis with infectious disease threats^35^ and foreshadowed the special challenge of responding to a public health crisis with divisive political undertones.

## The COVID Pandemic

By 2020, the United States had invested billions of dollars in public health emergency preparedness since the 9/11 and anthrax attacks. It was no surprise that the 2019 Global Health Security Index ranked the United States at the top of the world in readiness. SARS‐CoV‐2, however, would finally bring the latent weaknesses of US public health into full view.

The bright spots of the US response had their roots a decade earlier in investments in countermeasure development and procurement. Research sparked by the SARS outbreak helped National Institutes of Health researchers to quickly identify vaccine targets on the spike protein of the coronavirus.^36^ The search for better influenza vaccines spurred by pandemic fears accelerated the development of mRNA vaccine technology so that it was ready for the SARS‐CoV‐2 response. The government's authority to procure countermeasures (dating back to the 2006 Pandemic and All Hazards Preparedness Act) allowed the federal government to contract with private industry for the development of multiple vaccine candidates at once.^37^

Again, however, the United States was slow to share the benefits of its investments. President Trump pulled the country from the World Health Organization during the pandemic and limited participation in global efforts aimed at sharing vaccines.^38^ COVAX, an initiative launched by the Coalition for Epidemic Preparedness Innovations, the World Health Organization, and other global partners, had the mission of securing and distributing global access to vaccines, premised on the idea that all would be vulnerable until there was global protection. But high‐income countries including the United States secured vaccines for themselves first, and without adequate funding to secure advanced purchase commitments for vaccines at the same time high‐income countries did, COVAX was late to help countries unable to purchase vaccines themselves.

Other countermeasure‐related difficulties included a catastrophic failure at the CDC to rapidly develop a diagnostic test for SARS‐CoV‐2, limiting its ability to help individuals and monitor the pandemic during critical early months.^39^ There were major challenges in launching clinical research, which delayed the identification of optimal treatment regimens; other countries were more effective in completing informative clinical trials.^40^ The successful development of monoclonal antibodies was undermined by difficulty coordinating their deployment with the medical system.^41^

More profoundly, the pandemic exposed the inadequacy of the country's public health infrastructure. The magnitude and duration of the pandemic tested multiple foundational capacities of public health agencies; performance was uneven at best. Inadequate testing capabilities and antiquated laboratory information systems slowed or inhibited reporting and contributed to a lack of clarity about the epidemiology of the disease.^42^ Weaknesses in data systems and data‐reporting policies contributed to failures to track and respond to the health system's needs, contributing to preventable shortages of critical supplies and medications.^43^ Years of attrition in the public health workforce made the establishment and coordination of testing, tracing, and vaccination efforts difficult in many areas; for example, after‐action reviews in Missouri found a profound lack of confidence in the public health response in some areas.^44^

During the pandemic many public health agencies were unable to communicate with the public routinely and effectively. Underfunded and understaffed, health officials struggled to help the public understand what was happening and how to protect themselves. Misinformation and disinformation filled the void, amplified by divisive politics. Millions of Americans came to believe falsehoods about the vaccine and decided against vaccination.^45^ Meanwhile, relentless attacks led to the resignations of multiple health officials,^46^ and in more than 20 states, legislatures reduced health departments’ authority to take emergency actions to save lives.^47^ Trust in government has been an important determinant of mortality during the COVID‐19 pandemic.^48^ In the United States, trust in government fell to historically low levels.^49^

As is the case with other major emergencies, at every stage, SARS‐CoV‐2 exploited the divisions in society, by race, ethnicity, class, and geography. The underlying burden of preexisting health conditions, already reflecting the legacy of poverty and racism, put entire communities at greater risk.^50^ Working and living conditions made it impossible for millions to avoid the virus. Communities of color had less access to testing,^51^ treatment,^52^ and vaccination.^53^ Individuals with disabilities struggled to obtain basic services. Rural communities were more likely to believe misinformation and disinformation, less likely to be vaccinated, and—in combination with less access to health care facilities—more likely to die.^54^ Yet, facing this predictable situation, many public health emergency preparedness programs were slow to track disparities, launch collaborations, and pursue urgently needed policy changes. Public health agencies in many areas were forced to respond with new structures and programs to address these gaps.

As the disproportionate toll of COVID‐19 on the United States became evident, the pandemic provided a final lesson about how emergency preparedness rests on the degree of effectiveness of routine public health activities. Underappreciated in many international comparisons of rates of death from SARS‐CoV‐2 is the fact that the US population experiences far higher rates of chronic illness than the populations of many other countries.^55^ A key dimension of readiness for an emergency is the health of the population before disaster strikes, the primary focus of core public health activities.

## The Future of Public Health Preparedness

For the field of public health emergency preparedness in the United States, the long string of emergencies and outbreaks coming and going without significant reflection should come to an end. On the other side of a long‐overdue rethinking is a new conception of preparedness as fully integrated with global and national public health.

With respect to countermeasures, the field of public health emergency preparedness must commit itself to thinking beyond the syringe. The pandemic demonstrated the value of investing in basic countermeasure development and production. It also revealed the consequences of failing to take additional steps so that the products of scientific research quickly reach those in need. While the United States and other countries have committed themselves to the goal of developing pandemic vaccines in 100 days, much more work is needed to be able to scale manufacturing and develop reliable mechanisms to distribute vaccines to those most at risk. Also needed is enhanced capacity to execute rapid critical trials, including those with designs to simultaneously test and compare efficacy of different products. Countermeasure programs should also include research into their acceptance, particularly among those with deep distrust of the medical and public health authorities. This research should inform a modern and effective communications effort to explain the value of tests, vaccines, and medications to the public.

Global inequity in access to countermeasures compromises the world's ability to stop pandemics, fuels emergence of variant viruses, and contributes to global distrust and economic instability. As governments and funders turn to developing vaccines for diseases in the viral families most likely to cause serious epidemics, success will require equal attention to global manufacturing capacity and the strengthening of global public health infrastructure. To that end, the expansion of global childhood vaccination programs into vaccination programs across the life span is an essential preparedness investment.

For the United States, rethinking emergency preparedness programs demands a renewed commitment to public health broadly, recognizing that programs focused on emergency response depend on the foundation of core public health capacity to be successful. To be ready for emergencies, the United States needs a national public health system that delivers results every day, promotes health equity, and protects all Americans regardless of who they are and where they live.

A recent report from the Commonwealth Fund Commission on a National Public Health System provides a vision for such a national public health system.^56^ Led by former state, local, and federal public health officials, the commission made recommendations for federal leadership; for strengthening state, local, tribal, and territorial public health departments; for promoting collaboration with the health care system; and for enhancing community trust.

At the federal level, public health is the responsibility of HHS, where numerous agencies are involved in public health policy and funding, with efforts often overlapping and uncoordinated, and there is no single person or office accountable for public health or the infrastructure needed to support it. Over the past two decades, major investments in public health emergency preparedness have added to the confusion by creating new offices without a clear path for coordination among them. The Commonwealth Fund Commission recommended a new undersecretary for public health at HHS to lead efforts to rebuild the public health infrastructure and drive collaboration across federal agencies and health agencies across the country. At the top of this person's priority list should be modernizing public health data systems, which, as a recent report from the Robert Wood Johnson Foundation identified,^57^ can be accomplished with attention to equity at every stage.

Another critical task is to reinvigorate the workforce by providing sustainable federal funding and linking new staff to the achievement of foundational capabilities that should be required of every health department. An example is the opportunity to train local residents to serve as community health workers in health departments to support stronger partnerships and enhance trust. The Commonwealth Fund Commission's recommended investment of approximately $9 billion each year is a small fraction of the value to the national economy, given the enormous daily harm of preventable chronic illness^58^ and health disparities,^59^ let alone unnecessary suffering during a pandemic.^3^

Noting the importance of the health care response to the coronavirus pandemic, the Commonwealth Fund Commission also called for much stronger collaborations among health systems, community health centers, and public health agencies to tackle urgent challenges. This includes greater sharing of claims‐based data, electronic health record data, and data on the capabilities and resources of health care facilities, whose potential use to advance public health is today only rarely achieved. At the federal level, CMS should begin by providing timely, de‐identified data and maps of health conditions to state and local health agencies. CMS and other payors should also monitor critical information, such as receipt of vaccines, and monitor vaccine safety and effectiveness, sharing such information with federal and state public health agencies when needed.

What should emergency preparedness programs look like in well‐functioning public health departments? A primary goal should be to leverage the day‐to‐day opportunities to improve health to train for major emergencies. Every meningitis outbreak, localized surge in overdose, or spike in child or maternal mortality is an opportunity for assessment, planning, mobilization, and response. Emergency preparedness programs should bring together health departments with health systems to achieve clarity in who lives in the community and what their needs are, both every day and during emergencies. Dedicated preparedness staff should be fully engaged in this routine work, driving and measuring improvement in health and equity in order to build readiness for national disasters.

The Commonwealth Fund Commission ends its recommendations by calling for a renewed commitment for public health leaders to earn community trust. Echoing recommendations in a report by the Black Coalition Against COVID,^60^ the commission recommends greater efforts to counter misinformation; advocates for greater partnership, with funding, for community leaders and organizations to work alongside public health; and proposes additional steps to support ethics, transparency, and integrity.

Connecting public health emergency preparedness to public health foundational capacities broadly will be a political challenge. It is easier to talk about training exercises and stockpiles than about routine data systems and staffing. But preparedness programs without core capacities are like the horse without the cart or the race car without the track. To be successful in the future, the unstated assumption that preparedness rests on core public health capacity should be made explicit.

As data systems modernize, more accurate and timely understanding of novel threats can make the difference between responses that lumber to understand what is happening and those that quickly recognize the intersection of new challenges with health status, race/ethnicity, geography, and other relevant characteristics. As the public health workforce diversifies, communications capabilities improve, and community partnerships expand, trust will grow.

Public health agencies should collaborate with other sectors to understand and address root causes of poor health and inequity. Success in this effort will help communities become more resilient to stressors of all kinds, including public health emergencies. The Commonwealth Fund Commission called for the reestablishment of a national council to bring together federal departments across sectors to improve health. Local public health departments (and schools and programs of public health) should involve themselves with school systems, police departments, and housing authorities, seeking to use data effectively to illuminate new solutions to long‐standing problems. A healthier population will be less vulnerable to infectious diseases, extreme heat, and other threats of the 21st century. Every step toward a more just and equitable society is a step away from the brink of disaster.

For the first two decades of the 21^st^ century, the emphasis of public health emergency preparedness has been on the last two words—*emergency preparedness*. For the next decade and beyond, the focus should shift to the full phrase, starting with *public health*.
